# Is age kinder to the initially more able?: Yes, and no

**DOI:** 10.1016/j.intell.2011.10.007

**Published:** 2012-01

**Authors:** Alan J. Gow, Wendy Johnson, Gita Mishra, HALCyon Study Team, Marcus Richards, Diana Kuh, Ian J. Deary

**Affiliations:** a Centre for Cognitive Ageing and Cognitive Epidemiology, Department of Psychology, University of Edinburgh, Edinburgh, EH8 9JZ, UK; b MRC Unit for Lifelong Health and Ageing, Royal Free and University College London, WC1B 5JU

**Keywords:** Prior cognitive ability, Cognitive aging, Latent growth curve modeling, Longitudinal, Cohort study

## Abstract

Although a number of analyses have addressed whether initial cognitive ability level is associated with age-related cognitive decline, results have been inconsistent. Latent growth curve modeling was applied to two aging cohorts, extending previous analyses with a further wave of data collection, or as a more appropriate analytical methodology than used previously. In the Lothian Birth Cohort 1921, cognitive ability at age 11 was not associated with cognitive change from age 79 to 87, either in general cognitive ability, or in tests of reasoning, memory and executive function. However, data from the MRC National Survey of Health and Development suggested that higher cognitive ability at age 15 predicted less decline between ages 43 and 53 years in a latent cognitive factor from tests of verbal memory and search speed, and in search speed when considered separately. The results are discussed in terms of the differences between the cohorts and the interpretability of the analytical approach. Suggestions are made about when initial ability might be cognitively protective, and study requirements to bring about a clearer resolution.

## Highlights

► Identifying the predictors of cognitive aging is an area of increasing interest. ► It is unclear to what extent early cognitive ability might affect cognitive aging. ► We used data from 2 longitudinal studies of cognitive aging both with early life cognitive ability data. ► Early ability was protective against decline in midlife but not old age.

## 1 Introduction

Are those people with higher ability in early life more likely to retain their cognitive abilities with age? Longitudinal studies have demonstrated the stability in cognitive functions across the lifecourse; that is, the largest single predictor of the level of cognitive ability in later life is the level of cognitive ability from an earlier time (for example, Deary, Whalley, Lemmon, Crawford, & Starr, 2000; Richards & Sacker, 2003; Riley, Snowdon, Desrosiers, & Markesbery, 2005). However, it is important to distinguish this from the current question which concerns not how early cognitive ability predicts cognitive ability *level* at any given point in old age, but how it is associated with the amount of *change* in cognitive ability across time, or cognitive aging.

It has been said that age is kinder to the initially more able. Indeed, several research publications have based their titles on this statement (Christensen & Henderson, 1991; Deary, MacLennan, & Starr, 1998; Owens, 1959). The few existing empirical studies support contradictory conclusions. In some, those with higher ability in childhood or young adulthood decline less or at a slower rate when assessed some years or decades later (Bourne, Fox, Deary, & Whalley, 2007; Deary et al., 1998; Richards, Shipley, Fuhrer, & Wadsworth, 2004). However, in others, early ability level is unrelated to cognitive aging trajectories (Christensen & Henderson, 1991; Owens, 1959; Gow et al., 2011, 2008).

It is therefore unclear whether higher initial mental ability level is associated with a reduced risk of decline, or if there is no association. This uncertainty may be partly explained by the cognitive domains assessed across studies. Different cognitive abilities decline at different rates, and the age of onset of decline differs also (Hedden & Gabrieli, 2004). It is possible that domains of cognitive function are differentially affected by prior ability; the specific domains assessed in a given study may determine what effect, if any, is detected. Studies utilizing a battery of diverse mental tests allow an investigation of this but if a single test is used (or several tests drawn from a single cognitive domain), any conclusions can only apply to that narrow aspect of cognitive function. Studies addressing the ‘is age kinder’ question have covered a range of cognitive assessments. For example, Bourne et al. (2007) considered change in Raven's Progressive Matrices (a test of non-verbal reasoning). They reported that higher ability at age 11 years (assessed by an IQ-type test) predicted less decline from 64 to 66 years and, in a separate cohort, from 77 to 80 years. Because only Raven's was analyzed, it is not possible to extend the finding to other domains of cognitive ability, including, say, speed of processing or memory.

Other studies have considered a number of measures or domains simultaneously but independently (Christensen & Henderson, 1991; Richards et al., 2004), although an alternative approach has been to extract the common variance from a battery of cognitive tests as a marker of general cognitive ability (for example, Gow et al., 2008). In such analyses, the question is whether initial ability affects general cognitive aging, which obviously does not address potential differential associations of initial ability across cognitive domains. It is worthwhile, therefore, to consider the effect of prior cognitive ability on the aging of specific cognitive abilities *and* general cognitive ability, especially given that the latter has been shown to be the locus of much of the aging effect (Salthouse, 2004).

In addition to differences in the cognitive assessments, there is also the timing of when these are done. Some studies (Owens, 1959; Richards et al., 2004) considered adults in their 40s to 60s. It is possible that aging effects in the tests may not be detectable until later, or that ceiling effects may partially obscure effects in younger samples (Owens, 1959). Regression to the mean is also an issue to be considered when following individuals longitudinally, although this is more likely to lead to those of higher ability performing more poorly on subsequent occasions and those of lower ability subsequently performing better than expected, contrary to what has been reported by some studies (Bourne et al., 2007; Deary et al., 1998; Richards et al., 2004). This is not an exhaustive list of explanations for differences in results, and many have been discussed in detail elsewhere (for example, Deary et al., 1998). However, the choice of analytical technique is often overlooked as a contributor to cross-study discrepancies (Gow et al., 2008).

Interestingly, and notwithstanding the differences in study designs, it is important to reiterate that each was addressing the same key question: is age kinder to the initially more able? That inconsistent results are reported across studies and methodologies suggests there is no constructive replication. Constructive replication is the highest form of replication for a theory, and it has been suggested that most should be tested in this way (Lykken, 1968). In doing so, all conditions but the hypothesis being tested differ from one study to the next. The lack of consistency in results might suggest that initial ability does not affect cognitive aging across all ages and domains, so the question must be refined. There are circumstances in which protective effects of early ability are reported, and therefore potential mechanisms underlying this which need to be explained. To further investigate it is therefore important to consider the differences across studies by examining the nuances of the cohorts, tests and sampling ages, to allow a more thorough determination of when and under what circumstances early ability might and might not be protective.

There are a number of methods to analyze change over time and yet Reynolds, Gatz, and Pederson (2002) highlighted a lack of consensus in how best to examine cognitive aging trajectories. The authors suggested that the discrepancy across methods was due to differences inherent in the methods themselves, such that although they might be utilized for the same analytical purpose they are differentially suited to answering a given question of interest, and they cautioned: “when examining the relationship between baseline ability and rate of change, care ought to be taken when comparing studies that use different analytical methods” (Reynolds et al., 2002, p. 278).

Analyses in the ‘is age kinder’ debate have commonly employed linear regression or similar analytical methodology although Reynolds et al. (2002) suggested that random effects regression is preferable; it essentially uses a latent variable approach (estimating slope and intercepts) and is thus like the latent growth curve models which were used in the current analyses. Similarly, Christensen et al. (2001) suggested latent growth curve modeling as the most appropriate analytical method due to its focus on rate of change in individuals (rather than population averages as in linear regression, for example), and the possible inclusion of those with missing data. Furthermore, and importantly, latent growth curve modeling allows an investigation of factorial invariance in the cognitive measures applied across time. In essence, it is possible to consider whether latent factors from repeated waves of cognitive assessments are comparable; that they continue to assess the same constructs in the same way across assessments (Christensen et al., 2001). This is only applicable when multiple cognitive measures are used to create a latent variable reflecting general cognitive ability at each wave of assessment.

The issue of whether initial ability is protective against decline is related to the question of what protection education offers. In reviewing the evidence for a protective effect of education, Christensen et al. (2001) noted a number of limitations in the literature, including, but not limited to: the use of a single, broad measure of mental status (such as the MMSE, a basic screening tool) as the outcome, non-random attrition confounded with factors of interest (i.e. initial cognitive ability), inadequate sample sizes, and studies based on biased or unrepresentative age and ability compositions. These are equally applicable to the studies investigating whether initial ability protects against cognitive decline, although it is unlikely any single study can address all such limitations. There is a particular dearth of reported studies consisting of the following: a measure of cognitive ability from childhood or young adulthood followed by cognitive assessments on at least two, but preferably more, occasions some time later and most usefully in later life.

The variation across studies addressing the ‘is age kinder question’ is summarized in Table 1. Note that only those studies with measures of ability from childhood or young adulthood are presented. Those with baselines in mid- to late adulthood (Deary et al., 1998; Reynolds et al., 2002; Wilson et al., 2002) are not included, because participants in these samples may already have experienced age-associated decline. Also omitted are those studies using estimates or surrogates of prior ability (Christensen & Henderson, 1991; Christensen et al., 2001; Rabbitt, Chetwynd, & McInnes, 2003). Inconsistent results across studies might be due to small sample sizes and subsequent power to detect effects, age of participants at baseline assessments, the nature of the cognitive test(s), the ages at which participants were followed in later life, and the time between assessments. The analytical procedures employed might also affect the results reported as discussed above. The studies employing linear regression generally provide support for an effect of initial ability on later decline, but this technique might not be best suited to the question (Gow et al., 2008). However, examining Table 1 shows that the cross-study differences do not seem to show a consistent pattern which might explain the inconsistencies in findings. For example, Richards et al. (2004) began their follow-up at age 43, which is considerably younger than Bourne et al. (2007), where follow-up began in the 60s and 70s, yet similar results were reported. In addition, although the effects generally explain about 2% of the variance, even relatively small studies have shown this effect. We therefore sought to analyze new data, or reanalyze extant data with new methodology, as a first step towards trying to explain the pattern of differences.

### 1.1 The present study

Longitudinal data from 2 studies of cognitive aging were examined: the Lothian Birth Cohort 1921 (LBC1921) and the Medical Research Council (MRC) National Survey of Health and Development (NSHD; the British 1946 birth cohort). In both cohorts, cognitive ability data from childhood were available.

The NSHD data have previously been analyzed by linear regression (Richards et al., 2004). This suggested that on average, higher childhood cognitive ability (available at age 15 years) was associated with less decline in memory and search speed from age 43 to 53. Here, we applied growth curve modeling to this dataset for the first time. Since the previous comparison of regression and growth curve analyses in the LBC1921 (Gow et al., 2008), a third wave of cognitive ability data was collected and the previous analyses were extended to incorporate these new data. The principal aim was to identify if, and to what extent, early cognitive ability was related to the degree of individual cognitive decline using latent growth curve methodology.

## 2 Methods

### 2.1 Participants

#### 2.1.1 Lothian Birth Cohort 1921

The recruitment and testing of the Lothian Birth Cohort 1921 (LBC1921) at waves 1, 2 and 3 has been reported in detail previously (Deary, Whalley, & Starr, 2009; Deary, Whiteman, Starr, Whalley, & Fox, 2004; Gow et al., 2011, 2008). In summary, the individuals recruited into the LBC1921 were all born in 1921 and had taken part in the Scottish Mental Survey 1932 when aged 11 (N = 87,498). The LBC1921 study began in 1999 by identifying surviving participants of the Scottish Mental Survey 1932 from Edinburgh and surrounding areas. This initial wave of recruitment and testing ran until 2001, during which time 550 individuals (234 men and 316 women) were tested (Deary et al., 2004).

For the second wave, all LBC1921 participants, except those who had withdrawn or were known to have died, were invited to participate. Of the 454 participants invited, 321 were tested (145 men and 176 women) from 2003–05 (Gow et al., 2008). The third wave of testing ran from 2007–08. All LBC1921 participants who had completed both waves 1 and 2, excluding those who had withdrawn or were known to have died since wave 2, were invited to participate. Of the 268 participants invited, 196 participants were tested at the research clinic, and 11 participants were tested at home (97 men and 110 women: Gow et al., 2010). The recruitment and testing numbers are presented in Supplementary Table 1, including a breakdown of the attrition between and during assessments due to death or withdrawal.

The mean age of the LBC1921 when tested as children in the Scottish Mental Survey 1932 was 10.9 years (*sd* = 0.3). In late adulthood, the follow-ups occurred at mean ages of 79.1 (*sd* = 0.6: wave 1), 83.4 (*sd* = 0.5: wave 2), and 86.6 years (*sd* = 0.4: wave 3). For simplicity, these are referred to as ages 11, 79, 83 and 87 throughout.

#### 2.1.2 MRC National Survey of Health and Development

A full cohort description of the MRC National Survey of Health and Development (NSHD), also known as the British 1946 birth cohort, is available: Wadsworth, Kuh, Richards, & Hardy, 2006. The NSHD initially consisted of 5362 individuals all born in one week in March 1946 (Wadsworth, 1991; Wadsworth et al., 2006). Participants were from England, Scotland and Wales and were stratified by social class. Data were collected by clinical interview and questionnaire at regular intervals throughout childhood and adulthood; for this study, data from ages 11, 15, 43 (N = 3262) and 53 (N = 3035) were used. At the last wave, the sample was generally representative of the national population, and 49% were men (Wadsworth et al., 2006). Supplementary Table 1 also summarizes the key recruitment and testing numbers for the NSHD, and those lost to follow-up.

### 2.2 Procedure

In the LBC1921 and NSHD, the assessments consisted of a range of cognitive tests, collection of socio-demographic, lifestyle, and psychosocial information detailed medial histories, and physical testing. Only those variables relevant to the current analyses are described below. Further details can be obtained from: (Deary et al., 2009, 2004; Gow et al., 2011, 2008; Richards et al., 2004; Wadsworth, 1991; Wadsworth et al., 2006).

#### 2.2.1 Childhood cognitive ability

Participants in the LBC1921 completed a version of the Moray House Test (MHT) Number 12 when aged 11 years (Scottish Council for Research in Education, 1933). The test has a 45-minute time limit and the maximum possible score is 76. Participants in the NSHD completed an NFER-devised test of verbal and non-verbal ability at age 11 (Pidgeon, 1964) and were asked to select an appropriate word or shape to complete 80 different series. When aged 15, they completed the Alice Heim 4 test (AH4; Heim, 1970). The test consists of 130 verbal and non-verbal items, summed to give a general ability score. The raw age-11 MHT and age-11 verbal/non-verbal ability and age-15 AH4 scores were corrected for age in days or months respectively at the time of testing (age in days/months was entered as the independent variable in a linear regression with the relevant cognitive test as the dependent variable; the standardized residual was used as the age-corrected test score). In the LBC1921, the age-corrected MHT scores were then converted to the IQ score scale (thus by definition, the sample's childhood IQ had a mean of 100, and a standard deviation of 15).

#### 2.2.2 Adult cognitive ability

In the LBC1921, participants completed Raven's Progressive Matrices (RPM), Verbal Fluency (VF), and Logical Memory (LM) at ages 79, 83 and 87. Raven's Matrices consists of 60 items requiring non-verbal, inductive reasoning, and a 20-minute time limit was applied (Raven, Court, & Raven, 1977); Verbal Fluency is a measure of executive function requiring the generation of words starting with the letters C, F, and L (Lezak, Howieson, & Loring, 2004); Logical Memory is a subtest of the Wechsler Memory Scale–Revised (WMS-R: Wechsler, 1987) assessing verbal declarative memory.

In the NSHD, participants completed Verbal Memory and Search Speed at ages 43 and 53 (Richards et al., 2004). For Verbal Memory, participants were asked to recall a list of 15 words on 3 occasions, summed to give a total score. Search Speed required participants to complete a timed letter search, and the score was the number of letters scanned in 1 minute. Parallel versions were used at each wave to reduce practice effects.

For both cohorts, adult cognitive test performance was corrected for age in days at time of testing.

#### 2.2.3 Demographics

A number of demographic and psychosocial variables were included in the previous analyses with the LBC1921 and NSHD, and these are included here for consistency. At the first occasion of testing in the LBC1921 (age 79), participants were asked to provide the number of years spent in full-time formal education; their main occupation to allow social class coding (according to the 1951 Classification of Occupations (General Register Office, 1956), ranging from I (professional) to V (unskilled). Married women were assigned the higher of their own or husband's social class); whether they were current, ex- or never-smoker; and the frequency, amount and type of alcohol consumed per week to allow their average weekly alcohol unit intake to be calculated. This variable was capped at 49 units per week (6 outliers above this were recoded accordingly).

In the NSHD, smoking status at age 43 was recorded as current, ex- or never smoker. Average weekly alcohol consumption was calculated based on self-reported intake (spirits, wine and beer) converted to unit equivalents. Values above 70 units per week were capped to 70 (31 participants in total). Participants’ educational qualifications or training equivalents achieved by 26 years old were classified as: none, vocational only, ordinary secondary (O levels), advanced secondary (A levels), and degree level or equivalent. Age 43 occupational social class was classified according to the Registrar General, ranging from I (professional) to V (unskilled), with III (skilled) split into non-manual (IIIN) and manual (IIIM). For the current analysis, the highest social class of the household (participant or spouse) was used.

### 2.3 Statistical analyses

Descriptive analyses were conducted in PASW Statistics Version 17.0; growth curve modeling was carried out in Mplus Version 5.2. To examine the effect of childhood cognitive ability on cognitive aging (in the LBC1921 and NSHD), we implemented latent variable growth curve models which account for person-specific variability in cognitive aging. These analyses generate latent terms for intercept (level of cognitive ability) and slope (change in cognitive ability over time). The effect of the predictor variables on the intercept and slope can then be examined simultaneously. Under the assumption they were missing at random, participants with baseline data were included even if absent from subsequent waves using full information maximum likelihood. That is, even participants who withdrew from either study at any point, due to death or refusal, still provided some data for the analyses. FIML estimates tend to be less biased and more reliable than LD, even when the data deviate from MAR and are non-ignorable (Arbuckle, 1996).

#### 2.3.1 Measurement invariance

When multiple cognitive measures at a given wave were used to form a latent variable representing general cognitive ability, it was possible—and necessary—to examine measurement invariance. To assess measurement invariance, variations on the basic model were constructed. Firstly, a baseline model with all parameters allowed to vary freely was run; next, the same model was run with the factor loadings constrained equal; this was followed by a model in which the residual variances were constrained equal; finally, the intercepts were constrained equal. If applying these constraints results in deterioration of model fit at any step, measurement invariance cannot be demonstrated and it is not possible to interpret the slope parameter as change in the same construct measured in the same way across occasions (Meredith, 1993).

## 3 Results

### 3.1 Descriptives

Descriptive data for the NSHD are presented in detail in Richards et al. (2004); although this was a re-analysis of that same data, a summary is given in Supplementary Table 2. As the LBC1921 analysis represented a new wave of data collection, descriptive data for the cognitive tests are given in Table 2. The data in the first three columns are from the full sample at waves 1–3. Scores for both Verbal Fluency and Logical Memory appeared to be stable or increase slightly from ages 79 to 87. However, this was confounded by non-random attrition whereby those of highest baseline ability were more likely to return. The data in the latter half of the table are from the returning sample only. For all 3 tests, repeated measures ANOVA highlighted significant decline across the 3 waves [Verbal Fluency: F(1.929, 389.605) = 5.871, *p* = .003; Raven's: F(1.933, 377.009) = 85.268, *p* < .001; Logical Memory: F(1.833, 372.185) = 4.489, *p* = .014)].

Correlations between the tests completed at waves 1 and 2 have been discussed previously (Gow et al., 2008). The intercorrelations for the tests completed at wave 3 (age 87) ranged from .31 (*p* < .001) between Verbal Fluency and Logical Memory, to .47 (*p* < .001) between Raven's and Logical Memory. Performance on each test completed at age 87 was also highly correlated with performance on the same test at age 79 and 83 (for example, Raven's at age 87 correlated .74 with Raven's at 79, and .78 with Raven's at 83, both *p* < .001). Full details are available on request.

### 3.2 Testing for measurement invariance

Measurement invariance was examined by following the stepwise procedure described above, running from a model where the factor loadings, intercepts and variances were allowed to vary freely to one in which they were constrained equal across ages. Across the models in the LBC1921, the value of Chi-square increased from 15.568 (df = 15, *p* = .411) to 65.889 (df = 31, *p* < .001). However, compared to the first 3 models the final run had poorer fit statistics for RMSEA (.046, 95% C.I. = .030-.061, compared to .008, 95% C.I. = .000-.042 in the first model) and AIC (7178.763 compared to 7162.882). The failure of measurement invariance occurred at the final step requiring the intercepts to be constrained equal; for subsequent analyses, we used the final model that forced measurement invariance and address the implications of this in the discussion. In the NSHD, we were able to demonstrate measurement invariance through the stepwise procedure.

### 3.3 Modeling the influence of childhood cognitive ability on cognitive aging in the LBC1921

The LBC1921 latent growth curve models estimated the influence of age-11 IQ on the degree of cognitive change from age 79 to 83 and 87. There were two outcomes: cognitive ability *level* (intercept); and the *change* in cognitive ability across ages 79 to 87 (slope). Other potential contributors to the level of, and change in, late-life cognitive ability included: sex, social class, number of years of education, smoking status at age 79, and alcohol consumption at age 79. All variables were standardized prior to the modeling analysis.

**Fig. 1:**
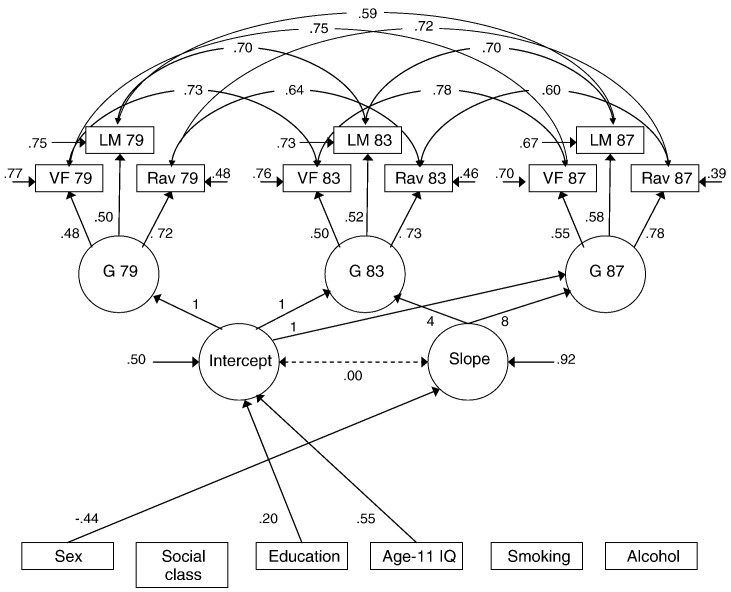
Latent growth curve model of the level and change in general cognitive ability over 3 waves of the Lothian Birth Cohort 1921. The model shows a latent general cognitive ability factor (G) at each age comprising VF = Verbal Fluency, LM = Logical Memory, and Rav = Raven's Progressive Matrices. Items in rectangles are measured variables, those in ellipses are latent traits. The numbers adjacent to the arrows leading from intercept and slope to the latent cognitive factors are fixed by the investigator (the 4 refers to the 4-year period between age 79 and 83, similarly for the 8). The other numbers—beside those arrows going from measured variables to latent traits, and beside arrows between latent traits—are parameters estimated by the program. These can be treated like standardized partial beta weights, and when squared give the proportion of variance shared by adjacent variables. For the covariates, paths and parameter estimates are only given for those paths that were significant *p* < .05, except the path between intercept and slope which is included for reference. All parameter estimates are standardized and given to two decimal places, except the path between intercept and slope which is the unstandardized value. For sex, the reference category was male. Education is the number of years in full-time formal education/qualifications attained; smoking status is defined as never, ex or current.

Fig. 1 illustrates the growth curve model of cognitive change in the LBC1921 with the three tests of cognitive ability forming latent general ability factors at ages 79, 83 and 87. The model fit well (Chi-square = 102.607, df = 76, *p* = .023, RMSEA = .025, 95% C.I. = .010-.037, TLI = .98 and CFI = .99), and the estimated correlation matrix is shown in Supplementary Table 3. Education and age-11 IQ were positively associated with the intercept (level of cognitive ability), with the largest contribution from age-11 IQ. Overall, the predictors accounted for 50.0% of the variance in the level of cognitive ability (*p* < .001). Sex was the only variable associated with the slope parameter, such that women showed greater decline, accounting for 8.2% of the variance in the slope. The model was also run with the covariates removed (only age-11 IQ remained) to ensure the lack of an early ability-slope association was not due to mediation by another covariate. The new model had poorer fit statistics and there was no association between age-11 IQ and slope. As would be expected with fewer covariates, the association between age-11 IQ and intercept increased (from .55 to .66).

The latent growth curve modeling was repeated considering each of the three cognitive measures separately and the results are summarized in Table 3 (full parameter estimates are available on request). Age-11 IQ was consistently the largest predictor of ability level (with path coefficients ranging from .24 for Logical Memory to .45 for Raven's). Education was associated with the level of Verbal Fluency and Raven's (better performance with higher education), alcohol consumption was positively related to Verbal Fluency level, and men were at an advantage on Raven's. None of the predictors were associated with the slope parameter in any of the models.

### 3.4 Modeling the influence of childhood cognitive ability on cognitive aging in the NSHD

**Fig. 2:**
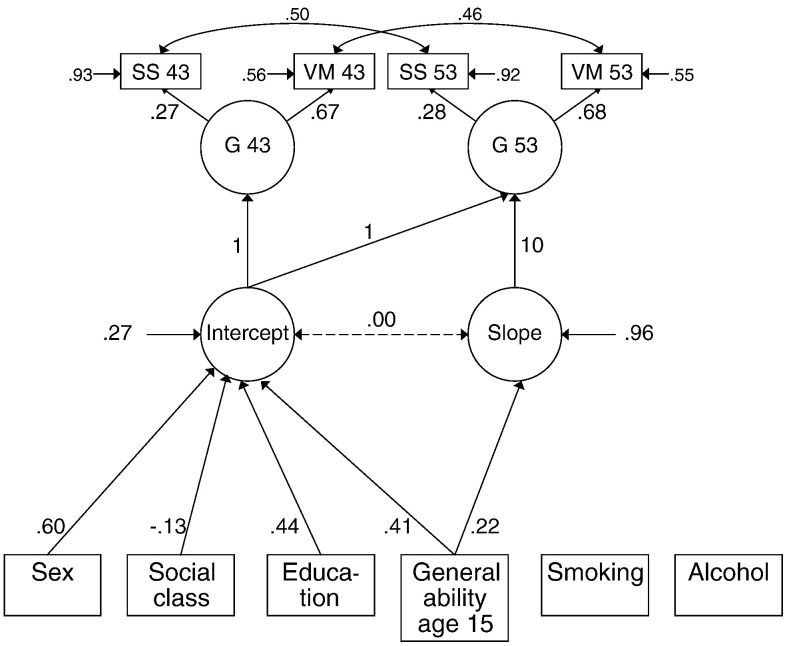
Latent growth curve model of the level and change in general cognitive ability over 2 waves of the MRC National Survey of Health and Development. The model shows a latent general cognitive ability factor (G) at each age comprising VM = Verbal Memory, and SS = Search Speed. See note for Fig. 1.

Similar latent growth curve models were created with the NSHD data, estimating the influence of age-11 or age-15 general ability on the level of cognitive ability, and the change from ages 43 to 53. Note, the analyses including age-15 general ability are described first as this represents a reanalysis of this data (Richards et al., 2004). The model with a latent general ability factor is illustrated in Fig. 2 with the estimated correlation matrix shown in Supplementary Table 4 (Chi-square = 70.208, df = 15, *p* < .001, RMSEA = .026, 95% C.I. = .020-.033, TLI = .97, CFI = .99). In the NSHD, sex (female advantage), social class (negative, although the direction of coding means that higher social class predicted higher intercept) and education (positive) were associated with the intercept (level of cognitive ability). Age-15 cognitive ability was positively associated with both the intercept and the slope; higher childhood cognitive ability predicted a higher level of cognitive ability at age 43, and less decline across the subsequent 10 years. Overall, the predictors accounted for 73.4% of the variance in the intercept and 4.2% in the slope, although the latter was not significant (*p* = .274). When age-15 IQ was the only covariate included, Chi-square was reduced to 4.973 (df = 5, *p* = .419). Although the association between age-15 IQ and intercept increased (.65, *p* < .001), the age-15 IQ-slope association was no longer significant (path coefficient .148, p = .068). This latter change should be judged in the context of a poorly fitting model.

When the cognitive tests were analyzed separately—summarized in Table 3, with parameter estimates available on request—sex, social class, education and age-15 cognitive ability were associated with the intercept for both Verbal Memory and Search Speed. In addition, alcohol consumption had a small, positive association with intercept in the Verbal Memory model. For Search Speed, smoking was negatively associated with the intercept. Sex and age-15 cognitive ability were associated with the slope. Those with higher childhood cognitive ability experienced less decline from 43 to 53 years. Women showed greater decline. In this model, there was also a negative association between the intercept and slope (unstandardized path coefficient -.05, *p* < .001). Although small, the association was significant. The direction of the association suggested that higher adult cognitive ability (intercept) was associated with greater subsequent decline (slope). Overall, the predictors accounted for 32.5% of the variance in the intercept but 0.4% in the slope for Verbal Memory; for Search Speed, the percentages of variance accounted for were 7.1% and 1.0%, respectively.

The NSHD models were repeated using the age-11 general ability score (instead of the age-15 score), summarized in Table 3, with parameter estimates available on request. In contrast to age-15 general ability, age-11 general ability was not related to decline in the latent general ability factor from 43 to 53 years (although it was associated with level). Age-11 general ability was however related to the change in Search Speed when analyzed separately (though not to level). The results were consistent with those previously when Verbal Memory was the outcome, whereby age-11 general ability predicted level but not change.

## 4 Discussion

The current analyses were focused on whether childhood cognitive ability was associated with cognitive aging. Data from 2 longitudinal studies were analyzed by latent growth curve methodology. In the Lothian Birth Cohort 1921 (LBC1921), there was no association between cognitive ability at age 11 and decline across 8 years (from age 79 to 87), either when 3 tests of cognitive ability were analyzed as a latent factor or individually. In the National Survey of Health and Development (NSHD), higher cognitive ability from age 15 was associated with less decline between ages 43 to 53 on general cognitive ability (a latent factor from Verbal Memory and Search Speed) and separately for Search Speed, but not Verbal Memory. When an earlier measure of cognitive ability was used in the NSHD, from age 11, only the associations with change in Search Speed remained. For both the LBC1921 and NSHD, higher childhood cognitive ability was associated with higher baseline adult cognitive ability.

The results from the LBC1921 are consistent with previous work from the cohort (Gow et al., 2011, 2008) suggesting that cognitive ability measured in childhood was not related to either 4-year change on the measures considered here, or 8-year change on the Moray House Test repeated at ages 79 and 87. Other studies have also reported no protective effect of early ability (Christensen & Henderson, 1991; Owens, 1959; Rabbitt et al., 2003). Taken together, these results suggest that age is no kinder to the initially more able. However, current and previous analyses of the NSHD data, and results from other studies run contrary to this (Bourne et al., 2007; Deary et al., 1998; Richards et al., 2004), indicating that higher childhood ability is associated with a more favorable cognitive aging trajectory. This was less apparent when the earlier measure of childhood ability was used which only remained associated with the 10-year change in a measure of processing speed. There is therefore a dearth of constructive replication (Lykken, 1968) of the general theory that early ability is always related to cognitive aging. It is important to consider the reasons for these discrepancies, such that the answer to the ‘is age kinder’ question might become distilled beyond a simple yes or no.

Firstly, it may be the results are not inconsistent, *per se*, or at least, not incompatible. The cohorts differ in terms of age composition, and it may be that prior ability is differentially associated with cognitive decline at different ages. For example, in the NSHD, a measure of processing speed declines more from age 43 to 53 in those of lower childhood ability. In the LBC1921, no childhood ability-cognitive aging associations were recorded between the ages of 79 and 87. It is possible that the age of the LBC1921 is such that the cognitive decline being observed is different to that in younger old age (through the 50s, 60s and 70s, for example). That is, the participants were recruited at a mean age of 79 and so were ‘selected’ in terms of those who had survived to that time. The LBC1921 are therefore likely to be healthier, of higher cognitive ability, and have a more ‘favorable’ status on a range of other socio-demographic confounders. The LBC1921 population were, therefore, the robust survivors of the once much more heterogeneous population that would have been available 20 years earlier (as discussed above). It is plausible the middle-aged population that included individuals who were destined for early frailty or even mortality experienced declines that more sensitively reflected the socio-economic differences (associated with their different levels of ability) than did the older surviving LBC1921. Contrariwise, if intelligence is a significant, but only partial, predictor of habits of living that tend to better health and delayed frailty and death we might speculate that the LBC1921 survivors were the sub-set of individuals in which individual differences in successful adherence to health habits *additional* to the variance associated with intelligence evened out survival. It is possible that after a certain age, people with different initial levels of intelligence decline at the same rate, but that earlier cognitive decline hits more selectively, varying with prior cognitive level. If there is an effect of childhood cognitive ability on the course of cognitive decline, it might be that the LBC1921 are beyond the age at which it is pertinent. The NSHD, on the other hand, were followed regularly throughout childhood and into adulthood and were generally population-representative at 53 years old (Richards et al., 2004). In terms of cognitive aging, however, the cohort is relatively young. It would be expected to see decline in processing speed over this period (which was the domain in which early ability predicted decline), but many other domains would fail to show discernable decrements until the late 50s and into the 60s (Hedden & Gabrieli, 2004; Schaie, 2005). [The finding of an effect on the processing speed measure is in contrast to the literature on the effect of education. Here, there appears to be consensus that higher levels of education do not protect against decline in speed measures (Christensen et al., 2001).]

The different birth years of the cohorts also mean they participated in the research within distinct lifecourse contexts: the NSHD were more likely to be in employment, with their daily routines structured around this although differing widely in terms of social, mental and physical engagement, whereas the LBC1921 were mostly retired by the time of the first assessment. Variation across cohorts in the continuity of daily tasks to earlier educational and other experiences might partly account for the difference observed in the results, although any effects of occupational and social stratification might be expected to persist into retirement, with implications for post-retirement wealth and health (Hyde & Jones, 2007).

The NSHD are currently undergoing repeat assessments (between 60–64 years old) and so it will be possible to investigate this further. In addition, there is a cohort related to the LBC1921—the Lothian Birth Cohort 1936—on whom childhood cognitive data are also available. This cohort has been assessed on a diverse battery of cognitive tests at age 70 (Deary et al., 2007) and is currently being followed up at age 73. These data will allow an examination of change in the 60s and 70s. However, our aim with the re-analyses of these datasets was also to stimulate others in this domain. It appears that age can be kinder to the initially more able, but much about this effect remains unclear. We would therefore urge those with relevant data to ask the question of it, using the methods described, directly addressing some of the issues raised here.

There was also the possibility that the age at which ‘early’ cognition is assessed is important. Assessment at a later age may be a more valid indicator of cognitive function. The reliability of this assessment would have implications for the likelihood of being related to later decline. Furthermore, when the NSHD data was analyzed using cognitive ability at 11, only decline in processing speed was associated with age-11 ability (whereas age-15 ability was also associated with decline in the latent factor of ability). Early measures of cognitive function may be differentially related to decline dependent on domain. There was no similar measure of processing speed in the current LBC1921 battery which may explain the lack of reported associations here. Given this, and the preceding cohort differences, a useful test would therefore now consist of multiple assessments of cognition throughout childhood and young adulthood (to examine what stage of development begins to predict later decline), followed some years or decades later by a broad battery of tests repeated over a number of waves (to examine if different domains are differentially affected by early ability), preferably through the 50, 60s and 70s (to examine whether decline through the decades is differentially affected by early ability).

When higher initial ability is protective against later decline (as suggested by the NSHD analysis), this may occur because declines are slower in those with higher ability, or the onset of declines is later (Christensen et al., 2001). Even when childhood ability is unrelated to the rate of change in later life, those with higher ability in childhood will enter their later years at a higher level—a consequence of the stability of cognitive ability across the lifespan (Deary et al., 2000; Gow et al., 2008). The mechanism through which early ability preserves later ability is important; it doesn't have to be something fixed to that higher level but something that people with initially higher level tend to do along the way. Those of higher initial ability are also more likely to move into safer and healthier working and social environments (Deary et al., 1998) which may both increase cognitive capacity via stimulation (participation in leisure pursuits or physical activity, for example: Richards, Hardy, & Wadsworth, 2003) and reduce the likelihood of exposure to cognitively detrimental factors (for example, cardiovascular disease and associated risk factors). This is similar to the notion of cumulative advantage/disadvantage, being “the systematic tendency for the interindividual divergence in a given characteristic (e.g., money, health, or status) with the passage of time” (Dannefer, 2003, p. 327). Richards et al. (2003) also suggested that the determinants of cognitive ability in childhood (genetic, uterine, home environment) might have lasting effects across the lifespan which could manifest as individual cognitive aging trajectories.

Investigating the effect of early ability on the rate of later decline is important because prior ability has been linked to the risk of developing vascular dementia (for example, McGurn, Deary, & Starr, 2008). Whether or not those of lower initial ability decline any faster than those of higher ability, given the stability of intelligence they will enter old age closer to any threshold for impairment and therefore be more likely to be classified accordingly with time. Furthermore, the findings from the NSHD suggest that prior ability may be related to the maintenance of ability through mid-adulthood, which would have consequences for the ability level at which individuals enter old age; that is, they would be doubly disadvantaged. In both cases, those of lower ability are at increased risk of reaching levels of cognitive function that preclude independent living before those of higher ability. Strategies to address cognitive decline would therefore be most beneficial if directed towards these higher-risk individuals, with early interventions being of the greatest potential benefit.

Growth curve methodology represents an appropriate tool for analyzing such data when considering latent factors of cognition (although the advantage of this approach is lessened with fewer data points or when the cognitive measures are examined separately). Christensen et al. (2001) noted:

“latent factors are superior measures of their underlying constructs due to their use of multiple indicators, evidence for factorial invariance and disattenuation of measurement error, there may be reason to prefer the findings of studies that rely on structural equation modelling” (p. 25).

Growth curve methods remove the bias from individual test variance which is not possible in linear regression analyses (Gow et al., 2008). It is also possible to examine measurement invariance in the cognitive assessments. Measurement invariance is assumed in the linear regression model, although it is necessary to show this is the case. Measurement invariance was shown to exist in the NSHD data but not the LBC1921. The latter finding makes interpretation of the slope parameter unclear. However, the dedifferentiation hypothesis, whereby cognitive domains become less distinct over time, might indicate that measurement invariance is less likely to be found in elderly cohorts as the structure of any general cognitive factor will change with age. As the NSHD are younger, the structure of a general factor of cognition might be more robust across waves (although the inclusion of only 2 cognitive tests on 2 occasions may also contribute to the finding of measurement invariance). Furthermore, growth curve modeling allows a simultaneous assessment of the associations between an array of predictor variables with level and change, and the inclusion of those supplying data at baseline but not later waves.

### 4.1 Strengths and limitations

While the possibility that the ages of the cohorts has resulted in the different findings is interesting, it is not possible to examine in detail without further data collection. The age of the participants when they completed the childhood tests of cognitive ability is potentially important; the LBC1921 were assessed when aged 11 and it is possible their performance was affected by pubertal stage. It is necessary to consider further reasons for the discrepant findings, which also comprise the general strengths and limitations of our analysis. We see these as refinements of the ‘is age kinder’ question, and hope there are extant datasets which can address these.

The LBC1921 may be underpowered to detect what appear to be small effect sizes. By the third wave of assessment (age 87), the sample numbered around 200 due to attrition, and consequently, variation in cognitive aging trajectories was small. On the other hand, the NSHD participants numbered in the thousands, and the study was better powered to detect such small effects. Although several times larger, the NSHD participants were somewhat younger and it is interesting to consider what cognitive changes might be expected in a relatively healthy group between the 40s and 50s.

Different cognitive tests were used across the cohorts, including those completed in childhood, which may partly account for the observed differences in results. Practice effects must also be considered, and it is possible that those of higher ability are more likely to benefit from repeated assessments (Christensen et al., 2001). The NSHD use parallel versions of the tests in order to reduce this potential confounder, and indeed, have 10 years between assessment waves. The LBC1921, however, were tested more frequently and the same tests were used on each occasion. A full assessment of cognition is ideal; different domains of functioning have been shown to decline at different ages and rates, and it has been suggested that education protects against declines in the crystallized but not fluid domains (Christensen et al., 2001). Although both cohorts included more than one cognitive domain, each was assessed by a single measure. Analyzing these as a latent cognitive factor was an attempt to address this weakness; however, it would be more appropriate to include multiple markers across a number of cognitive domains.

## 5 Conclusions

In summary, initial ability appears to be protective against later cognitive declines, although this may not happen across all stages of the aging process nor in all samples. The discrepancies may be due in part to methodological differences across studies, although it is possible that prior ability affects the trajectory of cognitive change during key periods (into the 50s and beyond but not the late 70s and 80s). It is suggested, however, that latent growth curve methodology is an appropriate analytical approach to this and related questions. Future analyses will benefit from using this approach. So, is age kinder to the initially more able? Yes. And no. That much we know; now it's time to refine the question to get a more detailed answer. Given the current analyses, it is important to carefully consider how and when early ability is measured; the timing of the later ability tests (stage of life); and the types of ability studied later.

The following are the supplementary materials related to this article.

Supplementary Table 1 Summary of recruitment, testing and attrition in the Lothian Birth Cohort 1921 and the MRC National Survey of Health and Development.

Supplementary Table 2 Mean (sd) cognitive ability test scores for the MRC National Survey of Health and Development.

Supplementary Table 3 Estimated correlations from the latent growth curve model of cognitive aging across 3 waves in the Lothian Birth Cohort 1921.

Supplementary Table 4 Estimated correlations from the latent growth curve model of cognitive aging across 2 waves in the MRC National Survey of Health and Development.

## Figures and Tables

**Table 1:** Summary of studies examining prior mental ability and later cognitive change.

Reference	Study	Baseline age and test	Follow-up (s)	Cognitive assessment	Analysis	Findings
Owens (1959)	Iowa State Army Alpha Study (N = 127, all male)	Age 19, Army Alpha Form 6	Age 50	Army Alpha Form 6	ANOVA and linear regression	No association between age 19 ability and change to age 50
Richards et al. (2004)	National Survey of Health and Development (N = 2058)	Age 15, Alice Heim 4	Ages 43 and 53	Memory and visual search speed	Linear regression	Age 15 ability predicted decline in memory and search speed over 10 years
Bourne et al. (2007)	Aberdeen Birth Cohorts of 1921 (N = 91) and 1936 (N = 349)	Age 11, Moray House Test	For 1921-born, ages 77 and 80 For 1936-born, ages 64 and 66	Raven's Progressive Matrices	Linear regression	Age 11 ability accounted for ~ 2% of the variance in Raven's change over 2–3 years
Gow et al. (2011, 2008)	Lothian Birth Cohort 1921 (N = 550)	Age 11, Moray House Test	Ages 79, 83 and 87	Moray House Test or composite from Raven's Standard Progressive Matrices, Verbal Fluency and Logical Memory	Linear regression and growth curve model	Age 11 ability accounted for ~ 1.4% of the variance in composite ability change over 4 years (regression); no association between age 11 ability and 4- or 8-year cognitive change (growth curve model)

*Note*. Only studies with a measure of cognitive ability in childhood or young adulthood are included.

**Table 2:** Mean (sd) cognitive ability test scores for the Lothian Birth Cohort 1921.

	Full sample			Returning sample (attended all 3 waves)		
	79	83	87	79	83	87
Verbal Fluency	40.0 (12.3)	39.8 (12.7)	40.0 (12.3)	42.1 (11.9)	40.9 (12.1)	40.1 (12.1)
Raven's	31.2 (8.8)	29.7 (9.2)	27.8 (9.2)	33.4 (8.3)	31.1 (8.3)	27.9 (9.1)
Logical Memory	31.6 (12.8)	33.0 (14.4)	32.8 (14.7)	34.8 (12.6)	34.7 (13.8)	32.8 (14.6)

*Note*. For the full sample, N = 543–548 at age 79, N = 317–320 at age 83, and N = 202–207 at age 87. For the returning sample, N = 203 for Verbal Fluency, 196 for Raven's and 204 for Logical Memory.

**Table 3:** Summary of domain-specific latent growth curve models in the Lothian Birth Cohort 1921 and the MRC National Survey of Health and Development.

		Model fit				Standardized path coefficients	
Cohort	Cognitive test	Chi-square	RMSEA	TLI	CFI	Intercept	Slope
LBC1921	Verbal Fluency	1.147 (p = .992)	.000 (95% C.I. = .000-.000)	1.03	1.00	Education = .17 Age-11 IQ = .35 Alcohol = .12	-
	Raven's Matrices	3.394 (p = .846)	.000 (95% C.I. = .000-.030)	1.02	1.00	Sex = −.35 Education = .13 Age-11 IQ = .45	-
	Logical Memory	5.416 (p = .609)	.000 (95% C.I. = .000-.045)	1.01	1.00	Age-11 IQ = .24	-
NSHD (age-15 general ability model)	Verbal Memory	0.000 (p < .001)	.000 (95% C.I. = .000-.000)	1.00	1.00	Sex = .36 Education = .29 Social class = −.09 Age-15 general ability = .28 Alcohol = .05	-
	Search Speed	0.000 (p < .001)	.000 (95% C.I. = .000-.000)	1.00	1.00	Sex = .34 Education = .12 Social class = −.04	Sex = −.12
						Age-15 general ability = .06	Age-15 general ability = .09
						Smoking = −.05	
NSHD (age-11 general ability model)	General cognitive ability factor	73.671 (p < .001)	.027 (95% C.I. = .021-.033)	0.97	0.98	Sex = .51 Education = .40 Social class = −.13 Age-11 general ability = .41	-
	Verbal Memory	0.000 (p < .001)	.000 (95% C.I. = .000-.000)	1.00	1.00	Sex = .30 Education = .26 Social class = −.08 Age-11 general ability = .29 Alcohol = .05	-
	Search Speed	0.000 (p < .001)	.000 (95% C.I. = .000-.000)	1.00	1.00	Sex = .33 Education = .13 Social class = −.04 Smoking = −.06	Sex = −.12
							Age-11 general ability = .06

*Note*. Latent growth curve models were run separately for the individual cognitive tests. Only the values for significant path coefficients between the intercept and slope are shown.
