# Supplementary material for: Is age kinder to the initially more able?: Yes, and no
Source: Intelligence. 2012 Jan;40(1):49–59. doi: 10.1016/j.intell.2011.10.007 (PMC3657153; doi:10.1016/j.intell.2011.10.007)
Supplement: Supplementary Table 1 — Summary of recruitment, testing and attrition in the Lothian Birth Cohort 1921 and the MRC National Survey of Health and Development. [file mmc1.pdf]

Supplementary Table 1

Summary of recruitment, testing and attrition in the Lothian Birth Cohort 1921 and the MRC National Survey of Health and Development

	Year	Age in years	Withdrawals before next invitation	Deaths before next invitation	Invited[a]	Successfully tested	Died	Lost contact	Temporary refusal	Withdrawal
LBC1921	1999-2001	79.1 (0.6)	-	-		550	-	-	-	-
	2003-05	83.4 (0.5)	40	69	454	321	10	13	94	16
	2007-08	86.6 (0.4)	3	51	268	207[b]	8	4	42	7
NSHD	1989	43	-	-		3262				
	1999	53	100	104	3673	3035	28	330	280	

Note. [a]For both cohorts, the number of participants invited to a given wave exceeds the number tested at the previous wave minus those dead or withdrawn between waves, as some participants were invited that had not completed the previous wave. [b]Thirty LBC1921 participants completed questionnaires but were unable to attend the clinic or receive a home visit, giving total N = 237; these participants are recorded in the table as temporary refusal as they are not included in the current analyses.

Supplementary Table 2

Mean (sd) cognitive ability test scores for the MRC National Survey of Health and Development

	Full sample		Returning sample (attended both waves)		
	43	53	43	53	
Verbal Memory	24.7 (6.4)	23.9 (6.3)	25.0 (6.3)	24.1 (6.2)	$t(2597) = 8.539, p < .001$
Search Speed	341.9 (76.2)	281.2 (76.2)	342.0 (76.2)	281.4 (75.7)	$t(2672) = 42.693, p < .001$

Note. For the full sample, N = 3059 for Verbal Memory and 3131 for Search Speed at age 43, and N = 2887 for Verbal Memory and 2933 for Search Speed at age 53. For the returning sample, N = 2598 for Verbal Memory and 2673 for Search Speed. The t-tests report significant decline across waves for both Verbal Memory and Search Speed.

Supplementary Table 3

Estimated correlations from the latent growth curve model of cognitive aging across 3 waves in the Lothian Birth Cohort 1921

	1	2	3	4	5	6	7	8	9	10	Mean	Variance
1. Age-79 cognition	1.00										-.06	.50
2. Age-83 cognition	.93	1.00									-.13	.55
3. Age-87 cognition	.78	.95	1.00								-.20	.74
4. Model intercept	1.00	.93	.78	1.00							-.06	.50
5. Model slope	-.07	.30	.57	-.07	1.00						-.02	.01
6. Sex	-.11	-.16	-.18	-.11	-.15	1.00					.59	.24
7. Social class	-.40	-.32	-.23	-.40	.16	.10	1.00				.01	1.00
8. Education	.49	.43	.35	.49	-.09	-.12	-.48	1.00			.00	1.01
9. Age-11 IQ	.67	.63	.54	.67	-.02	.02	-.42	.46	1.00		.03	1.03
10. Alcohol intake	.10	.05	.00	.10	-.13	-.25	-.15	.10	.04	1.00	-.01	1.03
11. Smoking status	-.01	-.02	-.03	-.01	-.03	-.12	.04	-.15	-.04	.16	.00	. 99

Note. The model intercept and age-79 cognition factor were equivalent in the model. The model slope expressed the change per year. For sex, the reference category was male (the mean of .59 reflected the fact that 59% of the sample was female). Smoking status at age 79 was defined as never, ex or current; Education was the number of years in full-time formal education. All continuous variables were standardized prior to analysis.

Supplementary Table 4

Estimated correlations from the latent growth curve model of cognitive aging across 2 waves in the MRC National Survey of Health and Development

	1	2	3	4	5	6	7	8	9	Mean	Variance
1. Age-43 cognition	1.00									.08	.07
2. Age-53 cognition	.93	1.00								.07	.08
3. Model intercept	1.00	.93	1.00							.08	.07
4. Model slope	-.13	.26	-.13	1.00						.00	.00
5. Sex	.24	.25	.24	.04	1.00					.48	.25
6. Education	.70	.69	.70	.03	-.09	1.00				.00	1.00
7. Social class	-.49	-.50	-.49	-.06	.01	-.47	1.00			.04	1.01
8. Smoking status	-.22	-.23	-.22	-.05	-.10	-.23	.18	1.00		.03	1.00
9. Alcohol intake	.07	.05	.07	-.04	-.33	.14	-.10	.17	1.00	-.02	.82
10. Age-15 general	.69	.74	.69	.18	-.01	.53	-.37	-.14	.11	.01	1.00

ability

Note. The model intercept and age-43 cognition factor were equivalent in the model. The model slope expressed the change per year. For sex, the reference category was male (the mean of .48 reflected the fact that 48% of the sample was female). Smoking status at age 43 was defined as never, ex or current. All continuous variables were standardized prior to analysis.
